# Predicting ESWL success for ureteral stones: a radiomics-based machine learning approach

**DOI:** 10.1186/s12880-025-01817-8

**Published:** 2025-07-04

**Authors:** Ran Yang, Dan Zhao, Chunxue Ye, Ming Hu, Xiao Qi, Zhichao Li

**Affiliations:** 1 Department of Radiology, Chongqing Western Hospital, No. 301, Huafu Avenue North, Jiulongpo District, Chongqing, 400050 China; 2 Department of Radiology, Second People’s Hospital of Jiu Long Po District, No. 318 Huayu Road, Jiulongpo District, Chongqing, 400052 China

**Keywords:** Extracorporeal shock wave lithotripsy, Ureteral stones, Radiomics, Machine learning

## Abstract

**Objectives:**

This study aimed to develop and validate a machine learning (ML) model that integrates radiomics and conventional radiological features to predict the success of single-session extracorporeal shock wave lithotripsy (ESWL) for ureteral stones.

**Methods:**

This retrospective study included 329 patients with ureteral stones who underwent ESWL between October 2022 and June 2024. Patients were randomly divided into a training set (*n* = 230) and a test set (*n* = 99) in a 7:3 ratio. Preoperative clinical data and noncontrast CT images were collected, and radiomic features were extracted by outlining the stone’s region of interest (ROI). Univariate analysis was used to identify clinical and conventional radiological features related to the success of single-session ESWL. Radiomic features were selected using the least absolute shrinkage and selection operator (LASSO) algorithm to calculate a radiomic score (Rad-score). Five machine learning models (RF, KNN, LR, SVM, AdaBoost) were developed using 10-fold cross-validation. Model performance was assessed using AUC, accuracy, sensitivity, specificity, and F1 score. Calibration and decision curve analyses were used to evaluate model calibration and clinical value. SHAP analysis was conducted to interpret feature importance, and a nomogram was built to improve model interpretability.

**Results:**

Ureteral diameter proximal to the stone (UDPS), stone-to-skin distance (SSD), and renal pelvic width (RPW) were identified as significant predictors. Six radiomic features were selected from 1,595 to calculate the Rad-score. The LR model showed the best performance on the test set, with an accuracy of 83.8%, sensitivity of 84.9%, specificity of 82.6%, F1 score of 84.9%, and AUC of 0.888 (95% CI: 0.822–0.949). SHAP analysis indicated that the Rad-score and UDPS were the most influential features. Calibration and decision curve analyses confirmed the model’s good calibration and clinical utility.

**Conclusion:**

The LR model, integrating radiomics and conventional radiological features, demonstrated strong performance in predicting the success of single-session ESWL for ureteral stones. This approach may assist clinicians in making more accurate treatment decisions.

**Trial registration:**

Retrospectively.

**Clinical trial number:**

Not applicable.

**Supplementary Information:**

The online version contains supplementary material available at 10.1186/s12880-025-01817-8.

## Background

Ureteral stones are a common urological condition with a rising global incidence, placing a substantial burden on healthcare systems [1]. Clinically, patients often present with pain and hematuria, which may be further complicated by obstruction or infection, significantly impairing quality of life [2]. Extracorporeal shock wave lithotripsy (ESWL), first introduced by Chaussy in 1980 [3], remains a widely used non-invasive treatment for ureteral stones. Compared with ureteroscopy, ESWL generally has a lower single-session stone-free rate, but offers advantages such as lower cost and fewer complications [4]. The success of ESWL is influenced by various factors, including stone-specific characteristics such as size, density, composition, and location, as well as patient-related anatomical parameters [5, 6]. Stones with multiple unfavorable factors are generally more suitable for ureteroscopy. Failure of ESWL may result in prolonged ureteral obstruction, recurrent symptoms, and the need for additional interventions. Therefore, a comprehensive and accurate assessment of ESWL efficacy is essential for optimal case selection and treatment planning.

In recent years, radiomics has emerged as a promising technique capable of extracting high-dimensional quantitative features from medical images, enabling the identification of lesion characteristics that are not visually discernible [7]. Meanwhile, the application of machine learning (ML) and artificial intelligence (AI) in the medical field has advanced rapidly, with various ML algorithms being employed for disease diagnosis and prediction [8, 9]. ML-based radiomics analysis tools have demonstrated promising capabilities in improving diagnostic accuracy, facilitating prognostic stratification, and guiding personalized treatment decisions [10, 11]. In the context of predicting ESWL success for ureteral stones, previous research has primarily focused on clinical variables and conventional computed tomography (CT) measurements [12, 13]. However, studies exploring the predictive value of radiomics in this setting remain limited. We hypothesize that integrating radiomics features of ureteral stones with conventional CT-derived measurements beyond stone characteristics, and applying multiple ML algorithms to develop and compare predictive models, may improve the accuracy of predicting the success of single-session ESWL.

To this end, this study aims to develop and validate a ML model that integrates radiomics and conventional radiological features to predict the success of single-session ESWL for ureteral stones. Such a model has the potential to provide streamlined and reliable decision support in clinical practice, thereby facilitating more accurate selection of optimal treatment strategies for patients.

## Methods

### Patient selection

This retrospective study analyzed data from 568 patients with ureteral stones treated at Chongqing Western Hospital between October 2022 and June 2024. All patients underwent noncontrast CT scans within two weeks prior to undergoing a single session of ESWL prior to treatment. The inclusion criteria were as follows: (1) a solitary ureteral stone on one side, with a maximum diameter between 4 and 20 mm; (2) availability of complete clinical, imaging, and follow-up data; and (3) no additional surgical or adjuvant treatments administered during ESWL. The exclusion criteria were as follows: (1) urinary tract malformations (e.g., horseshoe kidney, renal malrotation, or ectopic kidney); (2) a history of ureteral surgery (e.g., ureteroscopic lithotripsy or open surgery); (3) nephrostomy or double-J stent placement; and (4) incomplete clinical, imaging, or follow-up data.

**Fig. 1:**
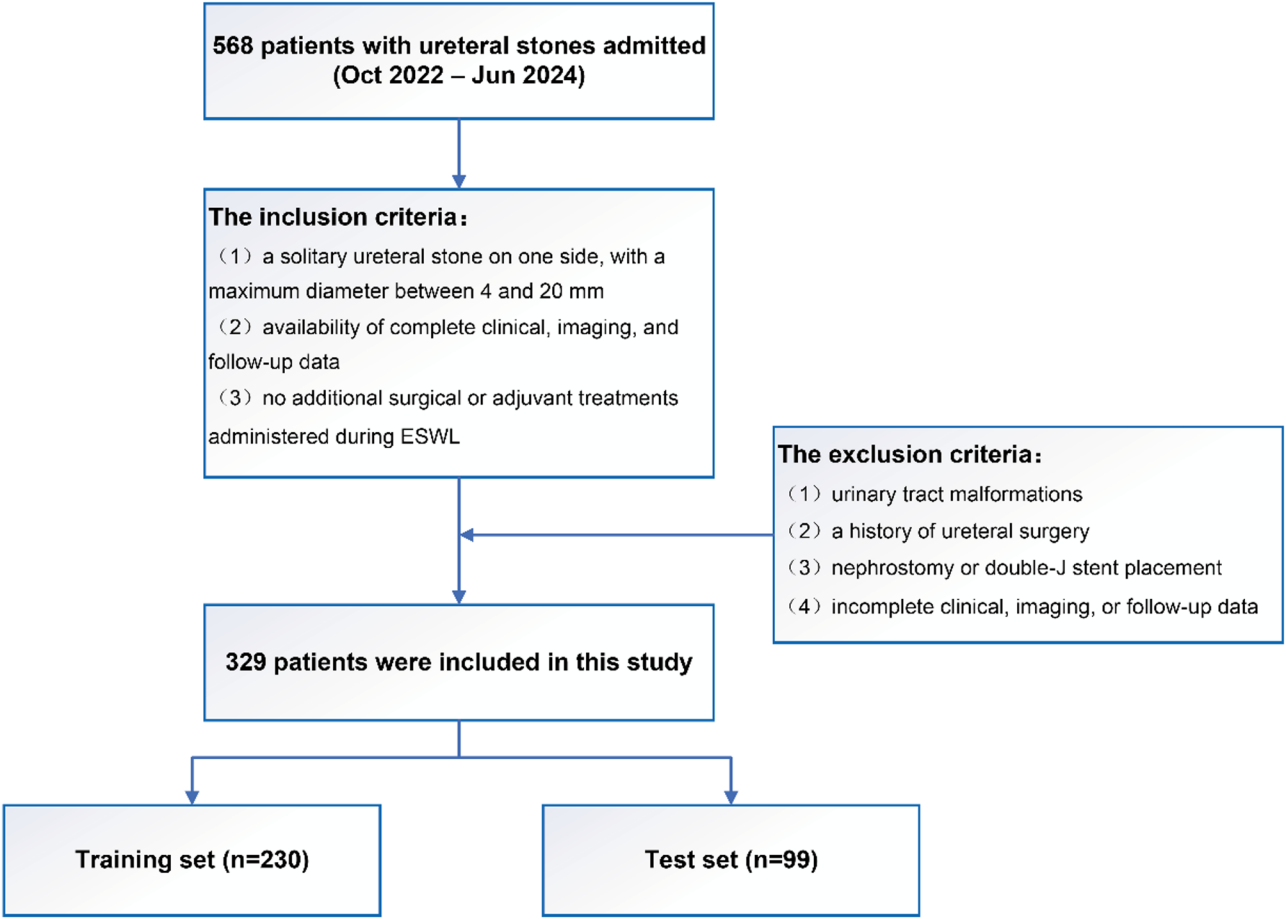
Patient recruitment process

On the basis of the inclusion and exclusion criteria, 329 patients were screened for analysis. The patients were subsequently randomized into training and test cohorts at a 7:3 ratio. The recruitment flowchart is shown in Fig. 1. The Ethics Committee of Chongqing Western Hospital reviewed the study design and determined that, due to its retrospective nature, formal ethical approval was not required. Therefore, the committee waived the need for formal ethics approval.

### ESWL protocol and follow-up assessment

All patients underwent ESWL via XYS. A single experienced urologist operated the SUI-6 C electromagnetic lithotripter (New Element Ltd., China). The procedure was performed without anesthesia, with stone localization guided by either X-ray or ultrasound. Patients were positioned in either the supine or prone position on the basis of stone location: upper ureteral stones were treated via the lumbar approach, whereas middle and lower ureteral stones were treated via the abdominal approach.

ESWL was performed via a stepwise energy-enhancement strategy, adjusted according to the patient’s tolerance. The shock wave frequency was set at 50–70 shocks per minute, with a maximum of 2,500 shocks per session. The peak voltage was limited to 15 kV to ensure safety and efficacy.

Follow-up evaluations were conducted two weeks after ESWL. Stone clearance was primarily assessed via kidney, ureter, and bladder (KUB) radiography or ultrasound. In cases where the findings were inconclusive, noncontrast CT scans were performed for confirmation. The imaging results were jointly assessed by an experienced radiologist and a urologist to ensure accuracy.

Patients were classified into two groups on the basis of the follow-up results: those with no residual stones or residual fragments ≤ 4 mm in diameter were categorized into the successful group. Conversely, patients requiring additional interventions (repeat ESWL or ureteroscopy) were classified into the unsuccessful group.

### CT image acquisition and clinical parameter collection

For CT imaging, patients were placed in a supine position with both arms raised above the head. A dual-source CT scanner (Siemens SOMATOM Drive, Siemens Healthcare, Germany) was used to perform an axial scan of the abdomen, covering the area from the diaphragm to the pubic symphysis. The scanning parameters were as follows: tube voltage, 120 kV; automatic tube current, detector collimation, 0.6 mm × 128; pitch, 0.6; rotation time, 0.5 s; reconstruction slice thickness, 1 mm; and reconstruction matrix, 512 × 512.

Clinical variables and conventional radiological features, including sex, age, affected side, stone location, maximum ureteral wall thickness (UWT _max_), skin-to-stone distance (SSD), ureteral diameter proximal to the stone (UDPS), and renal pelvis width (RPW), were collected.

The stone location was classified into three anatomical regions of the ureter: upper, middle, and lower. Stones located above the sacroiliac joint were categorized as upper ureteral stones, those located at the level of the sacroiliac joint were classified as middle ureteral stones, and those located below the sacroiliac joint were classified as lower ureteral stones.

Measurement definitions (Supplementary Fig. 1–4):

UWT _max_: The maximum thickness of the ureteral wall at the stone site, measured on axial images.

SSD: The distance from the stone to the skin, measured on axial images. For the lumbar approach, SSD was measured from the stone to the skin at the outer edge of the psoas major muscle or paraspinal muscle. The abdominal approach was measured as the shortest distance from the stone to the abdominal skin.

UDPS: The widest inner diameter of the ureter within 1 cm upstream of the stone, measured on axial images in a direction perpendicular to the ureteral lumen.

RPW: The transverse diameter of the renal pelvis at its widest level, measured on axial images perpendicular to its longitudinal axis.

All the measurements were independently performed by two radiologists with five years of experience. The intraclass correlation coefficient (ICC) [14] was used to assess interobserver agreement. If the ICC was ≥ 0.75, the mean value of the two measurements was used. If the ICC was < 0.75, a third radiologist with ten years of experience re-evaluated the measurements, and their result was taken as the final value.

### Region-of-interest segmentation and feature extraction

All the images were exported from the scanner in DICOM format and subsequently converted to NIfTI format via MRICroGL software (version 2.1.60). The NIfTI images were then imported into 3D Slicer (version 5.6.2), an open-source software for medical image visualization and analysis.

Two radiologists, each with five years of professional experience, independently segmented the regions of interest (ROIs). Two-dimensional ROIs were manually delineated along the lesion boundaries on each axial CT slice, and three-dimensional ROIs (volumes of interest, VOIs) were generated by stacking all two-dimensional ROIs.

Prior to feature extraction, the segmented images were resampled to a uniform voxel size of 1 mm × 1 mm × 1 mm. Radiomic features were then extracted via PyRadiomics (version 3.1.0a2), an open-source Python package. All feature extractions adhered to the guidelines of the Imaging Biomarker Standardization Initiative (IBSI) [15]. The features were derived from raw images, wavelet-transformed images, and Laplacian Gaussian-filtered images. A total of 1,595 radiomic features were extracted, as detailed in Supplementary Table 1.

**Fig. 2:**
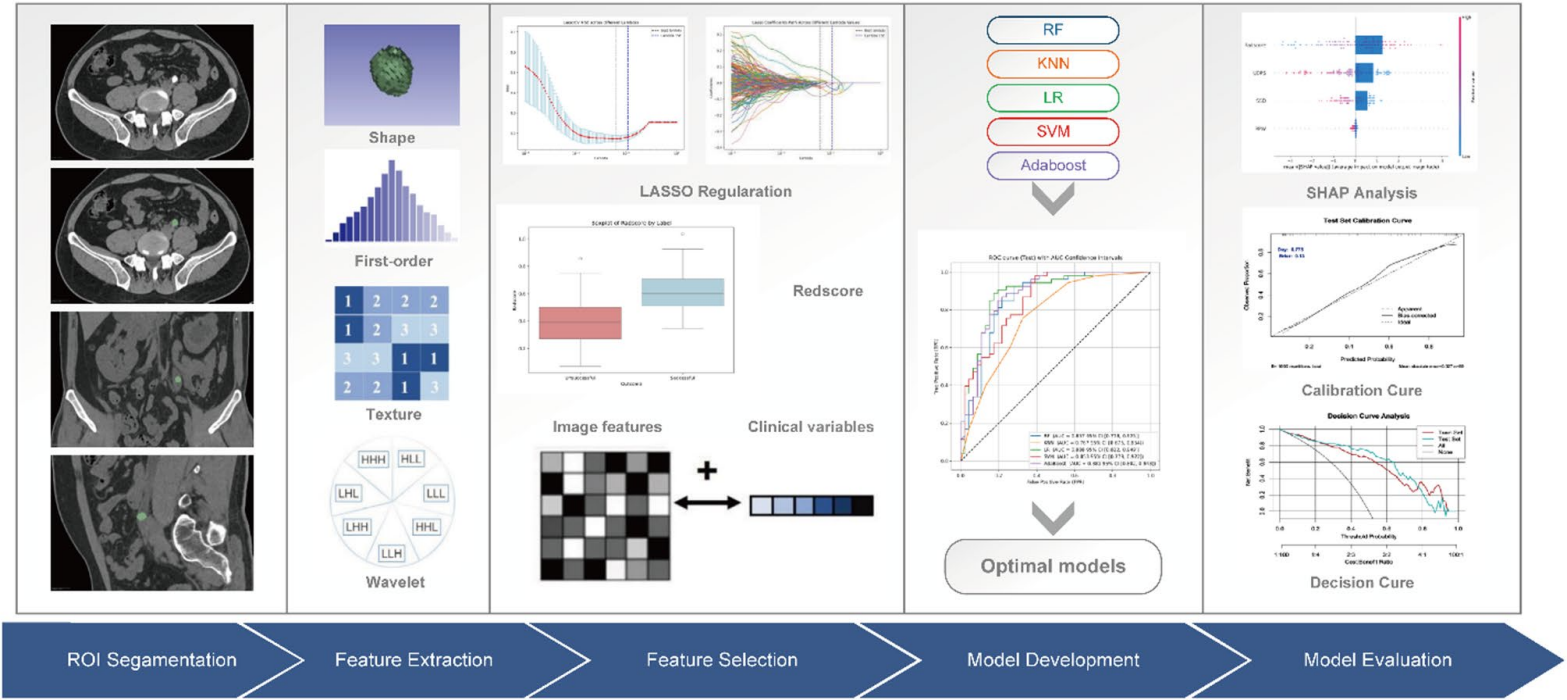
Technical workflow of this research

Definitions of texture parameters can be found at the PyRadiomics official site (https://pyradiomics.readthedocs.io/en/latest/features.html). The technical workflow of this study is illustrated in Fig. 2.

### Radiomics feature selection

To ensure the stability of the radiomic features, 50 samples were randomly selected and reanalyzed after a two-week interval. Two radiologists independently delineated the ROIs and extracted features following the same protocol. Interobserver reliability and intraobserver repeatability were evaluated via interclass and intraclass correlation coefficients (ICCs). Features with good reproducibility (ICC ≥ 0.75) were retained for further analysis. The retained features were Z score standardized to normalize differences in scale. Feature selection was performed via the least absolute shrinkage and selection operator (LASSO) algorithm, with 10-fold cross-validation used to determine the optimal penalty coefficient (λ) and select nonzero features. The radiomics score (Rad-score) for each patient was computed via the following formula: Rad-score = Σ (feature value × feature weight) + b₀ (intercept).

### ML model development and evaluation

Five ML algorithms were employed for model training: random forest (RF), k-nearest neighbors (KNN), logistic regression (LR), support vector machine (SVM), and adaptive boosting (AdaBoost). Stratified 10-fold cross-validation was performed on the training set to enhance the stability of model evaluation, with the process repeated 10 times to maximize data utilization and reduce randomness in data partitioning. A grid search was performed to optimize the hyperparameters of each algorithm and identify the optimal parameter combination.

All the ML models were implemented via the scikit-learn Python library (version 1.5.1). The optimal model was selected on the basis of the area under the receiver operating characteristic curve (AUC) evaluated on the test set. Shapley additive explanations (SHAP) analysis was performed to elucidate feature contributions and the model’s decision-making process [16]. The model’s diagnostic performance was further evaluated via calibration curves and decision curve analysis. Finally, a nomogram was constructed to provide a visual representation of the model’s predictions.

### Statistical analysis

Data analysis was performed via SPSS (version 27.0), R (version 4.4.2), and Python (version 3.9). Normally distributed data are expressed as the means ± standard deviations and were compared via the independent samples t test. Nonnormally distributed data are expressed as medians (interquartile ranges) and were compared via the Mann‒Whitney U test. Categorical data are expressed as frequencies (percentages) and were compared via the chi-square test. The performance metrics of all the ML models, including the area under the curve (AUC), accuracy, sensitivity, specificity, precision, positive predictive value (PPV), negative predictive value (NPV), and F1 score, were evaluated. Differences in AUC values were compared via the DeLong test. Model interpretability analysis was performed via the Python package SHAP (version 0.43.0). A P value < 0.05 was considered statistically significant.

## Results

### Patient characteristics

This study included 329 patients with ureteral stones, 168 of whom were successfully treated with ESWL and 161 of whom were not. Patients were randomly divided into a training set (230 cases: 115 successful, 115 unsuccessful) and a test set (99 cases: 53 successful, 46 unsuccessful) at a 7:3 ratio. Baseline data analysis revealed no significant differences in variables between the training and test sets (*P* > 0.05, Table 1). In the training set, univariate analysis revealed significant differences in UDPS, SSD, and RPW between patients with successful and unsuccessful treatment. Conversely, age, sex, affected side, stone location, and UWT max were not significantly different (*P* < 0.01, Table 2). Patients who achieved successful single-session ESWL had a smaller UDPS, shorter SSD, and narrower RPW.

**Table 1:** Comparison of baseline characteristics between the training and test sets

Variable	Training set (*n* = 230)	Test set (*n* = 99)	*P*-value
Successful (n) / Unsuccessful (n)	115/115	53/46	0.556^**a**^
Age [years, M (IQR)]	42(33,53)	42(33,53)	0.730^**b**^
Sex [n (%)]			0.533^**a**^
Male	157(68.3)	71(71.7)	
Female	73(31.7)	28(28.3)	
Side [n (%)]			0.779^**a**^
Left	127(55.2)	53(53.5)	
Right	103(44.8)	46(46.5)	
Location [n (%)]			0.183^**a**^
Upper	119(51.7)	62(62.6)	
Middle	33(14.3)	12(12.1)	
Lower	78(33.9)	25(25.3)	
UWT _max_ [mm, M (IQR)]	2.5(1.7,3.2)	2.5(1.7,3.2)	0.912^**b**^
UDPS [mm, M (IQR)]	5.3(4.4,6.5)	5.2(4.4,6.3)	0.841^**b**^
SSD [cm, M (IQR)]	10.3(9.1,11.4)	9.9(8.5,11.6)	0.490^**b**^
RPW [mm, M (IQR)]	14.3(11.8,18.1)	14.6(11.9,18.3)	0.632^**b**^

*UWT*_*max*_ maximum ureteral wall thickness, *UDPS* ureteral diameter proximal to stone, *SSD* skin-to-stone distance, *RPW* renal pelvis width

^a^ Statistical analysis performed using chi-square test

^b^ Statistical analysis performed using Mann–Whitney U test

**Table 2:** Univariate analysis of clinical and conventional CT radiological features of the training set

Variable	Successful (*n* = 115)	Unsuccessful (*n* = 115)	*P*-value
Age [years, M (IQR)]	40(32,52)	44(34,54)	0.346^**b**^
Sex [n (%)]			0.887^**a**^
Male	78(67.8)	79(68.7)	
Female	37(32.2)	36(31.3)	
Side [n (%)]			0.507^**a**^
Left	61(53.0)	66(57.4)	
Right	54(47.0)	49(42.6)	
Location [n (%)]			0.956^**a**^
Upper	60(52.2)	59(51.3)	
Middle	17(14.8)	16(13.9)	
Lower	38(33.0)	40(34.8)	
UWT _max_ [mm, M (IQR)]	2.5(1.7,3.1)	2.6(1.8,3.2)	0.741^**b**^
UDPS [mm, mean ± SD]	4.6 ± 0.9	6.4 ± 1.5	<0.01^**c**^
SSD [cm, mean ± SD]	9.8 ± 1.6	10.7 ± 1.7	<0.01^**c**^
RPW [mm, M (IQR)]	13.3(10.9,16.2)	15.6(13.1,20.1)	<0.01^**b**^

*UWT*_*max*_ maximum ureteral wall thickness, *UDPS* ureteral diameter proximal to stone, *SSD* skin-to-stone distance, *RPW* renal pelvis width

^a^ Statistical analysis performed using chi-square test

^b^ Statistical analysis performed using Mann–Whitney U test

^c^ Statistical analysis performed using independent-samples T test

### Feature selection and construction of the Rad-score

**Fig. 3:**
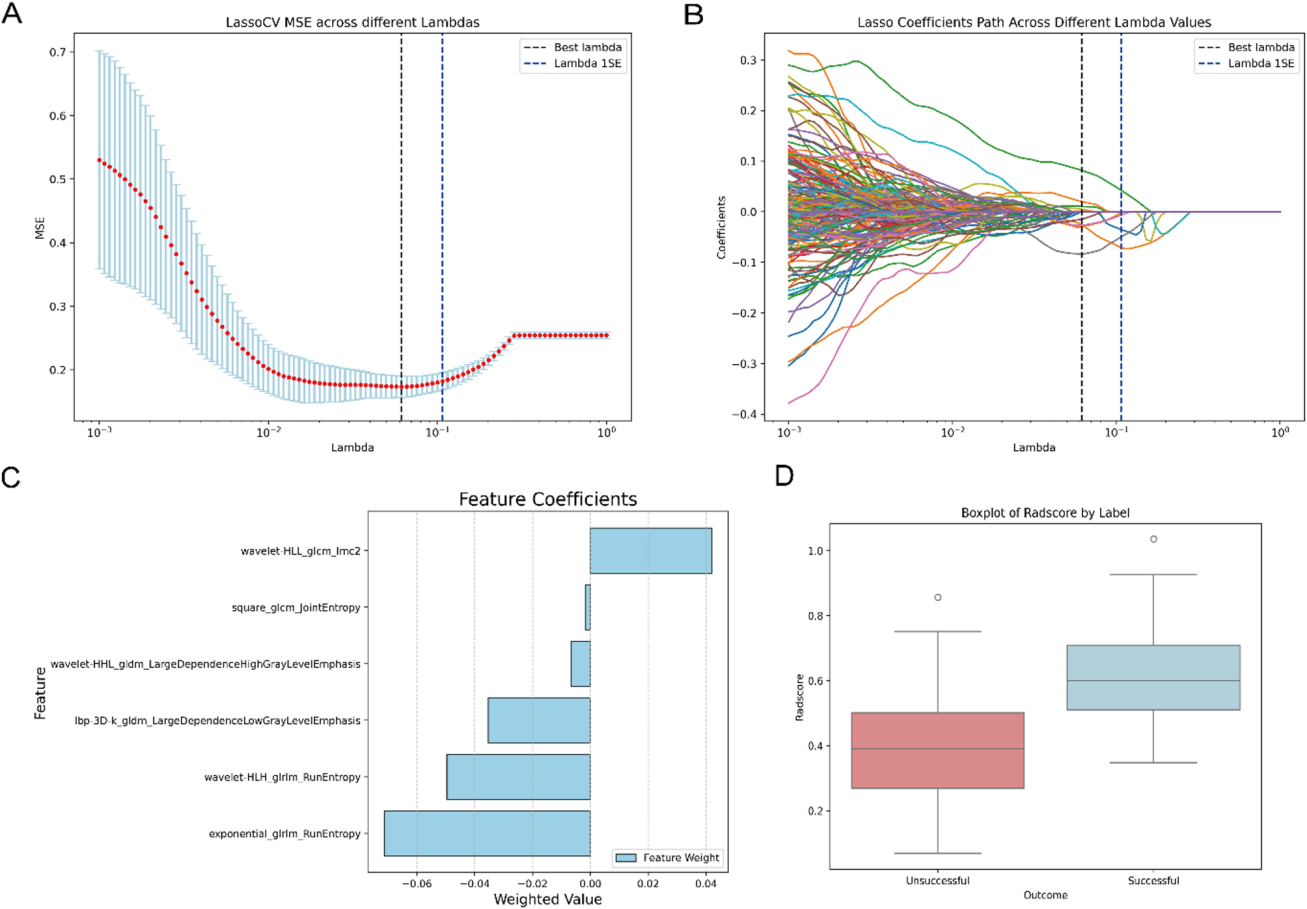
**(A)** Feature selection via the LASSO regression model. The lambda parameter was tuned via cross-validation, with the black dashed line on the left indicating the optimal lambda value that minimizes the evaluation metric (λ _min_) and the blue dashed line on the right indicating the lambda value within one standard error of the optimal value (λ _1se_). **(B)** The coefficient distribution plot illustrates the changes in feature coefficients as the regularization parameter lambda varies. Features with nonzero coefficients were retained for the final model. **(C)** Selected features and their corresponding weights. **(D)** Comparison of Rad-scores between the successful and unsuccessful groups in the training set

A total of 1,093 robust features (ICC ≥ 0.75) were identified. Using LASSO regression with 10-fold cross-validation, six features associated with ESWL efficacy prediction were selected (Fig. 3A–C). These features included two features from the gray-level run-length matrix (GLRLM), two from the gray-level co-occurrence matrix (GLCM), and two from the gray-level dependence matrix (GLDM). The Rad score was calculated via a specific formula (Supplementary Fig. 5). The results indicated that the Rad-score in the successful group was significantly greater than that in the unsuccessful group within the training set (*P* < 0.01, Fig. 3D).

### ML model performance comparison

**Fig. 4:**
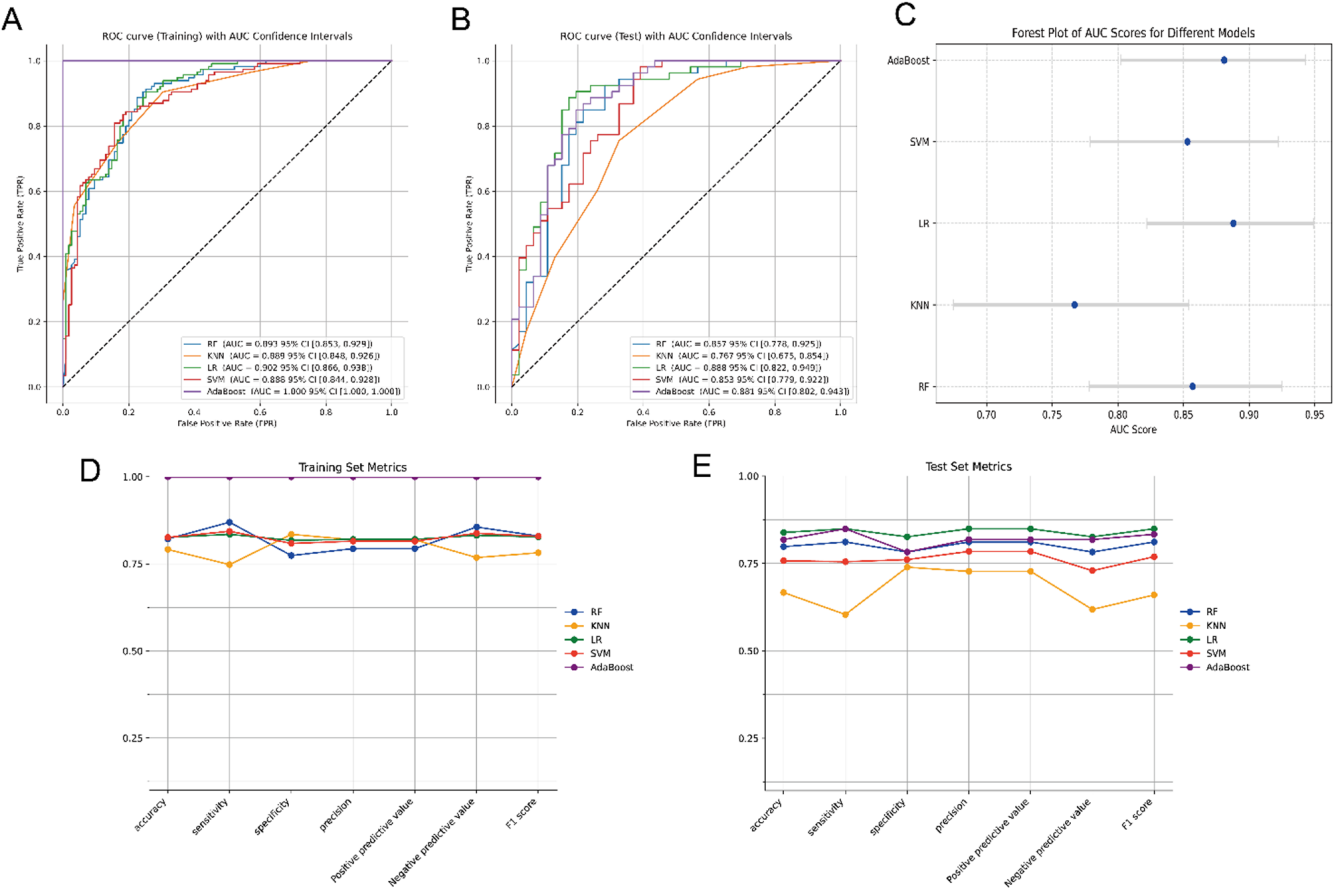
Performance and comparison of five predictive models. **(A)** ROC curve for the training set. **(B)** ROC curve for the test set. **(C)** Forest plot of the AUC values for the five models. **(D)** Evaluation metrics for the training set. **(E)** Evaluation metrics for the test set

Five ML algorithms—RF, KNN, LR, SVM, and AdaBoost—were used to integrate UDPS, SSD, RPW, and the Rad score for model development. In the training set, the AUC (95% CI) for each model was as follows: RF, 0.893 (0.853–0.929); KNN, 0.889 (0.848–0.926); LR, 0.902 (0.866–0.938); SVM, 0.888 (0.844–0.928); and AdaBoost, 1.000 (1.000–1.000) (Fig. 4A). In the test set, the AUCs (95% CI) were as follows: RF, 0.857 (0.778–0.925); KNN, 0.767 (0.675–0.854); LR, 0.888 (0.822–0.949); SVM, 0.853 (0.779–0.922); and AdaBoost, 0.881 (0.802–0.943) (Fig. 4B). Figure 4C presents a forest plot comparing the AUCs of the five ML models, whereas Supplementary Fig. 6 provides a Delong test heatmap comparing the AUCs between the models. Additionally, the accuracy, sensitivity, specificity, precision, positive predictive value, negative predictive value, and F1 score were calculated and compared for each model (Fig. 4D, E; Supplementary Table 2). Both the LR and AdaBoost models demonstrated strong performance on the test set, with no statistically significant difference in AUC, as evaluated by the Delong test. However, the LR model outperformed AdaBoost across all the evaluation metrics, demonstrating better generalizability. In contrast, the AdaBoost model exhibited potential overfitting in the training set. Consequently, the LR model was selected as the optimal model because of its superior overall performance and reliability.

### The LR model

The LR model was identified as the optimal predictive model and underwent further evaluation. The logistic equation is as follows:

logit(P) = 3.516005 + 8.201397 * Rad score − 0.703513 * UDPS − 0.365321 * SSD − 0.012403 * RPW.

**Fig. 5:**
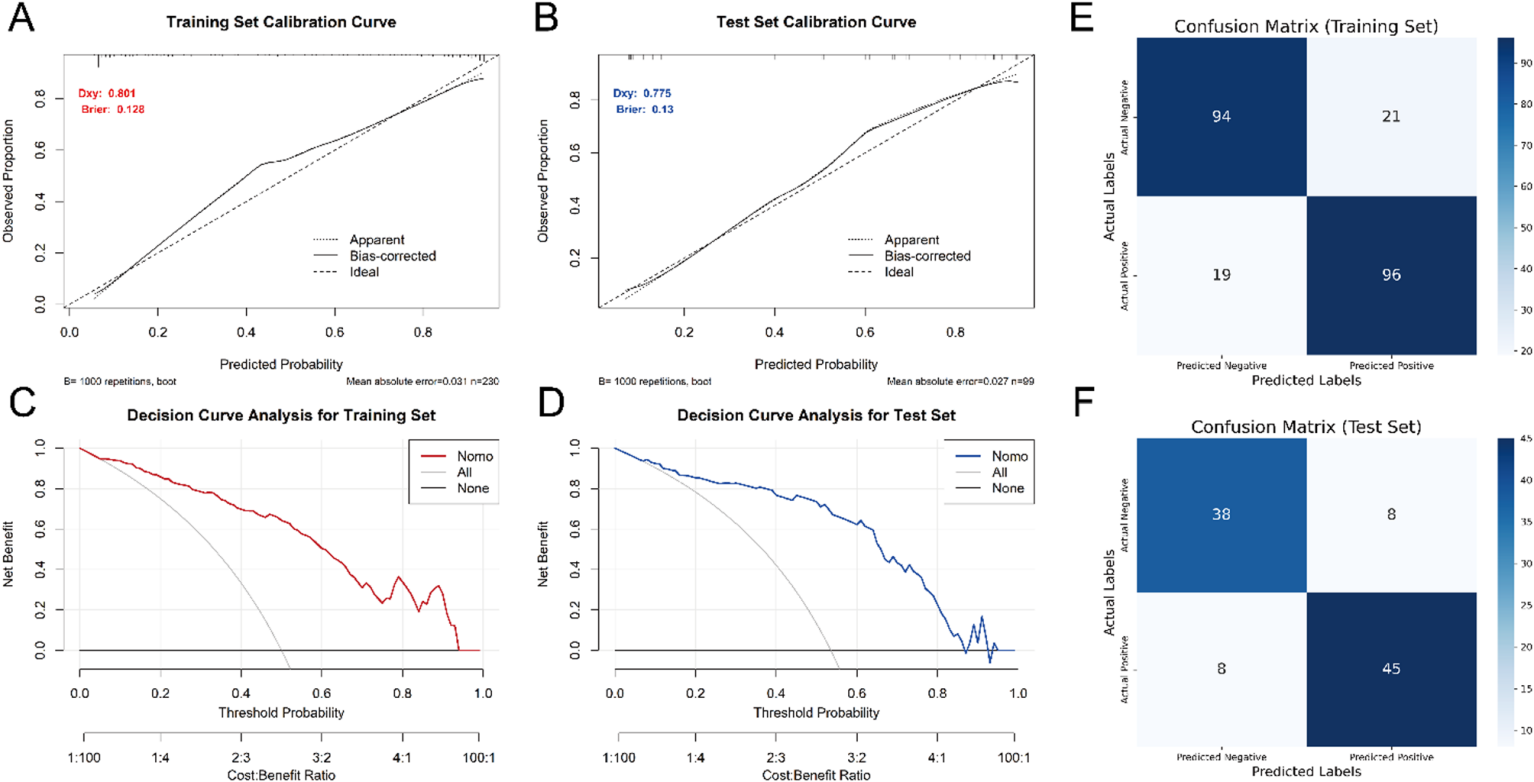
Comprehensive evaluation of the logistic regression model. **(A)** Calibration curve for the training set. **(B)** Calibration curve for the test set. **(C)** Decision curve analysis for the training set. **(D)** Decision curve analysis for the test set. **(E)** Confusion matrix for the training set. **(F)** Confusion matrix for the test set

The model’s predictive accuracy and calibration were evaluated via calibration curve analysis in both the training and testing datasets. In the training set, the calibration curve indicated high predictive accuracy, with a Somers’ D coefficient of 0.801, reflecting excellent discriminative ability. The Brier score was 0.128, highlighting the model’s reliability in prediction (Fig. 5A). In the testing set, the model retained strong discriminative power, albeit with a slight decline in predictive accuracy (Somers’ D coefficient = 0.775, Brier score = 0.13) (Fig. 5B).

Decision curve analysis for the training set (Fig. 5C) demonstrated that the model provided a significantly greater net benefit than did the baseline strategy when the threshold probability ranged from 0.1 to 0.95. Similarly, in the testing set (Fig. 5D), the model achieved good net benefit, within the threshold probability range of 0.1–0.85.

The confusion matrix results revealed differences in the model’s performance across datasets. In the training set (Fig. 5E), the model correctly identified 94 true negatives and 96 true positives while misclassifying 21 false positives and 19 false negatives. This corresponded to a true positive rate (sensitivity) of 83.5% and a true negative rate (specificity) of 81.7%. In the testing set (Fig. 5F), the model correctly identified 38 true negatives and 45 true positives but misclassified 8 false positives and 8 false negatives, resulting in a true positive rate of 84.9% and a true negative rate of 82.6%.

### SHAP-based model interpretability analysis

**Fig. 6:**
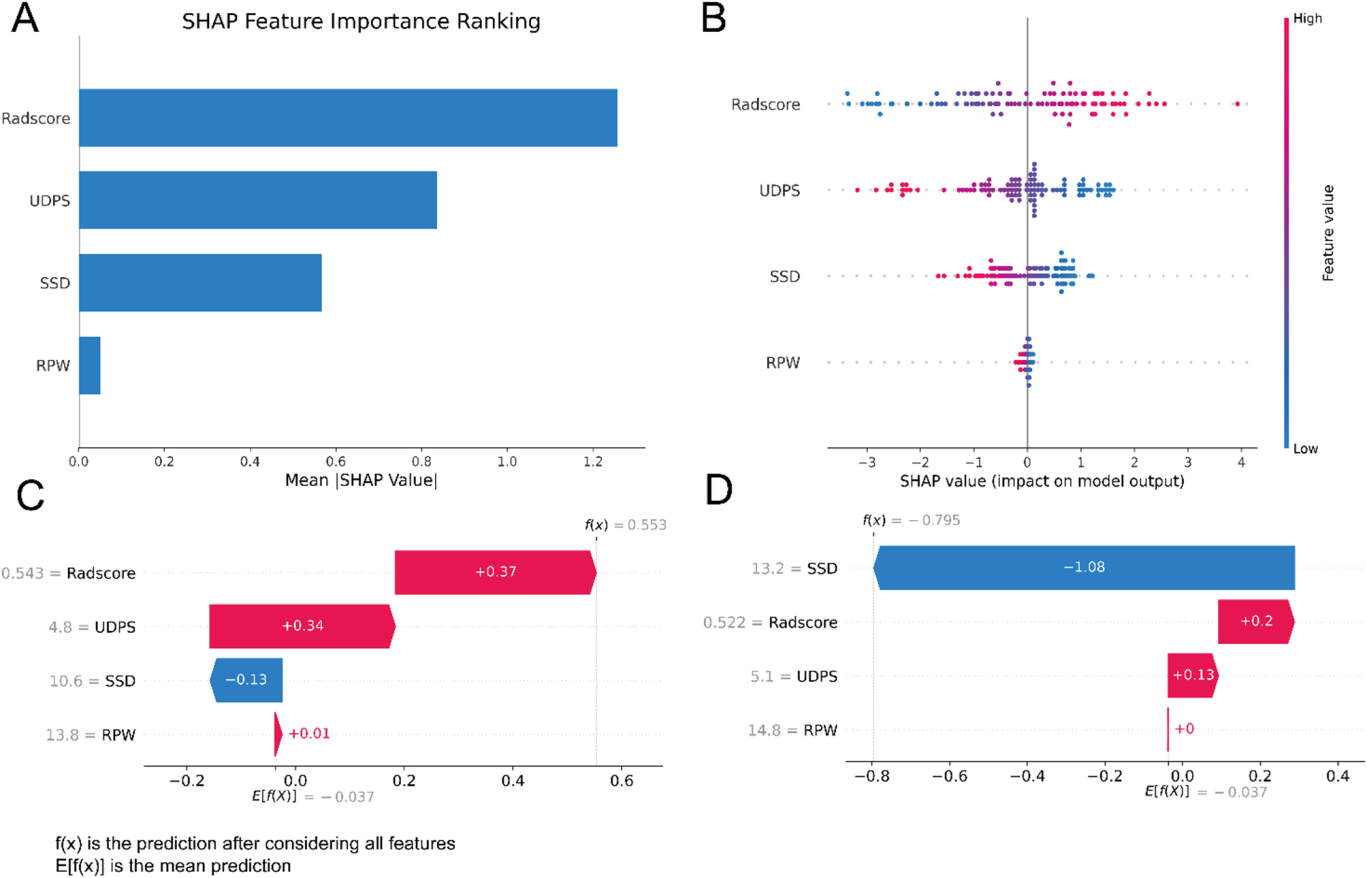
Interpretability analysis of the LR models. **(A)** Importance ranking plot of features in the LR model. **(B)** SHAP dendrogram showing feature importance, correlations, and distributions in the LR model. **(C**,** D)** SHAP-based interpretability analysis of two representative samples

SHAP analysis was performed to interpret the contributions of individual features and the model’s decision-making process. Figure 6A illustrates the hierarchical importance of each feature in the prediction model, with features ranked on the vertical axis in descending order of importance and the horizontal axis representing the average SHAP value. The analysis reveals that the Rad score has the most significant effect on the model’s predictions. Figure 6B provides a detailed visualization of this ranking, where each point represents a sample. The color gradient, from blue to red, indicates the magnitude of the feature value. The vertical axis ranks the features by importance, and the plot also illustrates the correlation and distribution of feature values with their corresponding SHAP values. To provide deeper insights into the model’s decision-making process at the individual level, interpretability analysis was conducted on two representative samples (Fig. 6C and D). By visualizing the SHAP values for these samples, the influence of each feature on the model’s predictions for specific instances becomes more evident.

### Construction of the nomogram

**Fig. 7:**
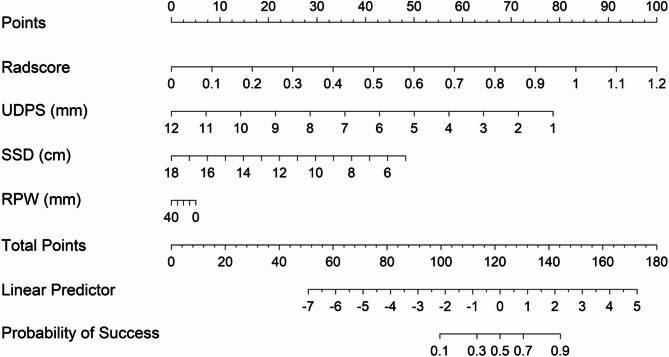
A nomogram based on the established LR model was constructed to predict the single-session success rate of ESWL for ureteral stones

On the basis of the established LR model, a nomogram was constructed to visually estimate the single-session success rate of ESWL for ureteral stones, with UDPS, SSD, RPW, and the Rad score as predictive variables (Fig. 7).

## Discussion

The technical core of this study is radiomics and ML. ML is a key branch of AI that uses algorithms to learn underlying patterns and relationships between variables from data without explicit programming. Typically, machine learning algorithms optimize model parameters using training data to capture statistical patterns or regularities. After training, the model can predict or classify new, unseen data [17, 18]. Radiomics is an automated and reproducible technique that enables standardized extraction of high-throughput features from medical images. Radiomics-based methods have been widely used in clinical practice, including tumor staging, early diagnosis, and disease differentiation [19–21]. Although radiomics research has primarily focused on oncology applications, its potential in non-oncologic fields is also increasingly recognized. In urinary stone research [22], its applications include predicting surgical efficacy [23], analyzing stone composition [24], and distinguishing stones from phleboliths [25]. Combining the data processing capabilities of machine learning algorithms with the microscopic analysis of lesions provided by radiomics is the highlight of this study.

In this study, we employed five ML algorithms—RF, KNN, LR, SVM, and AdaBoost—to develop classifiers for predicting the treatment outcome of ESWL for ureteral stones. Each algorithm has unique advantages and limitations. RF, an ensemble learning method, performs classification by aggregating predictions from multiple decision trees through majority voting. It handles complex nonlinear relationships well and is robust to overfitting but may struggle with high-dimensional sparse data [26]. KNN is a simple algorithm based on distance metrics that classifies samples by comparing their proximity to training samples. While effective for small datasets, it is less efficient for large, high-dimensional data [27]. LR is a linear model that predicts class probabilities by fitting linear relationships. It is easy to implement and interpret but performs poorly on complex nonlinear problems [28]. The SVM classifies data by finding the hyperplane that maximizes the margin. It is effective for high-dimensional and nonlinear data, leveraging kernel functions to map data into higher-dimensional spaces. However, SVMs have high computational costs and require careful parameter tuning [29]. AdaBoost improves accuracy by combining multiple weak classifiers, such as decision stumps. While it performs well with nonlinear data, it is sensitive to noise and prone to overfitting [30]. We selected these algorithms for their complementary strengths in handling different data features and compared their performance to identify the best model for predicting the outcome of extracorporeal lithotripsy.

Our results indicate that the LR and AdaBoost models outperform the other models, warranting further analysis. In the training set, AdaBoost achieved a perfect AUC of 1.0, whereas LR attained an AUC of 0.902, demonstrating AdaBoost’s superior performance during training. However, this exceptional result suggests a potential risk of overfitting. In the test set, AdaBoost’s AUC decreased to 0.881, which was slightly lower than LR’s value of 0.888, indicating that AdaBoost exhibited slightly weaker generalization on unseen data. In terms of accuracy, LR also performed marginally better (0.838 vs. 0.818), further reflecting its robustness in the test set. Overall, while AdaBoost demonstrated outstanding performance on the training set, signs of overfitting may have contributed to its decline in test set performance. In contrast, LR maintained more consistent results across both datasets, suggesting stronger generalization ability and greater suitability for applications requiring stable performance.

Previous studies have also highlighted the predictive efficacy of LR and AdaBoost in ESWL outcome assessment. For example, Zhang et al. [31]. developed an LR-based nomogram to predict postoperative treatment outcomes of symptomatic ureteral stones following extracorporeal shock wave lithotripsy during the COVID-19 pandemic, which demonstrated strong predictive performance. Moghisi et al. [32]. conducted a large sample study comparing six nonlinear machine learning algorithms for predicting ESWL outcomes in patients with urinary stones, with the results indicating that AdaBoost outperformed other models. Consistent with our findings, both studies highlighted the predictive efficacy of LR and AdaBoost in ESWL outcome assessment. Given the stable performance of the LR model in this study, along with its advantages—including simplicity, ease of interpretation, efficient training, and greater readability and usability in clinical applications—we selected it as the optimal model. However, it is important to acknowledge the potential of the AdaBoost algorithm, particularly in handling complex and high-dimensional data in large-scale studies, which warrants further exploration in future research.

The identification of key variables that influence the success of ESWL for ureteral stones is crucial. In the selection of radiomics features, we employed the LASSO algorithm with tenfold cross validation to screen the feature sets corresponding to two commonly used λ parameters (λ _min_ and λ _1se_, where λ _min_ is the optimal λ value that minimizes the evaluation metric and where λ _1se_ is the λ value within one standard error of the optimal value). This process resulted in the selection of 14 features (Supplementary Fig. 7) for λ _min_ and 6 features (Fig. 3C) for λ _1se_. Given the small sample size and to simplify and stabilize the model, reduce noise interference, and enhance interpretability, we opted to use the 6 features under the λ _1se_ value to construct the Rad score.

Among these, the feature exponential-glrlm-Run-Entropy is derived from the GLRLM, which measures the complexity and uncertainty of the continuous grayscale variations in the image. It is particularly sensitive to high grayscale values. A higher value indicates a more complex grayscale pattern and a richer image structure. wavelet-HLH-glrlm-Run-Entropy refers to the run entropy of the GLRLM after wavelet transformation (HLH filter). lbp-3D-k-gldm-Large-Dependence-Low-Gray-Level-Emphasis represents the large dependency areas emphasizing low grayscale values after applying the local binary pattern (LBP). wavelet-HHL-gldm-Large-Dependence-High-Gray-Level-Emphasis highlights large dependency areas emphasizing high grayscale values after wavelet transformation (HHL filter). Both of these features, which are based on the GLDM, are related to the uniformity and grayscale distribution within the image. Square-glcm-joint-entropy is a joint entropy feature calculated from the GLCM after a square transformation. It measures the uncertainty in the joint distribution of grayscale levels across image pixels, with the square transformation accentuating the effect of high grayscale values. A higher value reflects a more complex joint distribution between pixel pairs with differing grayscale levels, indicating a more random or uneven texture. Finally, wavelet-HLL-glcm-Imc2 is a radiomic feature derived from the wavelet transform and GLCM and is used to measure the texture complexity and information relevance within the image, particularly in regions with a mixture of high-frequency and low-frequency information. A high Imc2 value suggests a blurred lesion boundary, whereas a low Imc2 value indicates a sharp contrast, manifesting as a clear boundary. Notably, in addition to texture and intensity features, the feature set under the λ _min_ parameter also includes two shape features: original-shape-minor-axis-length and original-shape-maximum2D-diameter-slice. The former represents the minor axis length of the lesion in three-dimensional space, whereas the latter describes the maximum diameter within a single two-dimensional slice. These features reflect the correlation between stone size and the success of ESWL; however, their feature weights are relatively low.

Previous studies have shown that stone characteristics—such as composition, density, and size—are closely associated with the stone-free rate after ESWL [5, 33, 34]. These studies typically rely on non-contrast CT images and assess parameters such as the mean or maximum Hounsfield unit, as well as the long and short diameters of the stones. However, different measurement methods may yield varying results. Radiomics analysis can help reduce this variability through its standardized processes of ROI delineation and feature extraction. Moreover, it allows for a more detailed, microscopic-level depiction of stones, enabling more accurate characterization of their features. Our study also demonstrates the correlation between intrinsic stone characteristics and the ESWL stone-free rate, as reflected in grayscale intensity, texture, and size features.

Most patients with ureteral stones present with acute onset and severe pain, often requiring emergency intervention, making rapid treatment recommendations crucial. When selecting clinical variables and conventional radiological features for this study, we prioritized data that could be quickly obtained from clinical assessments and easily measured on noncontrast CT images. In this study, UDPS, SSD, and RPW were significantly associated with ESWL success for ureteral stones, whereas age, sex, affected side, stone location, and UWT _max_ were not significantly different. As SSD increases, the ultrasound travel distance increases, reducing energy transmission and diminishing stone fragmentation capability. While SSD is widely recognized as a factor influencing ESWL success, some controversy remains [6], likely due to differences in measurement methods. In our study, SSD was measured along the actual lithotripsy path: for the lumbar approach, SSD was defined as the distance from the stone to the skin at the outer edge of the psoas or paraspinal muscle; for the abdominal approach, SSD was the shortest distance from the stone to the abdominal skin. In contrast, other studies often take SSD measurements at 0°, 45°, and 90°, either averaging these values or selecting the 45° measurement as representative. UWT, proximal ureteral diameter (PUD), and hydronephrosis are frequently studied in the context of ureteral stone impaction [35, 36]. Some studies have reported a significant correlation between these factors and ESWL success, particularly for proximal ureteral stones [37–39]. However, in our study, UWT did not show a statistically significant association, which may be attributed to the fact that most patients presented with acute onset, likely in the early stages of the disease. Since our study included ureteral stones at all locations and PUD was measured primarily at the ureteropelvic junction—relevant to proximal ureteral stones but less reflective of the lithotripsy environment for mid- and distal ureteral stones—we opted to use UDPS. The UDPS was defined as the widest inner ureteral diameter within 1 cm upstream of the stone on axial images. Hydronephrosis assessment methods vary across studies. In this study, we employed RPW, defined as the transverse diameter of the widest part of the renal pelvis on axial images. This approach streamlined the evaluation process by eliminating the need for complex measurements and grading. Moreover, by ensuring that SSD, UDPS, and RPW can all be rapidly measured on axial images, our method provides a quick and practical assessment framework for real-world clinical applications.

In this study, we employed the SHAP method to provide both global and local explanations for ML models, enhancing their transparency and visual interpretability. Kaidi Gong et al. [40]. observed that SHAP demonstrated superior consistency and performance compared with traditional weight-based explanation methods, exhibiting greater stability across different models. Similarly, Yasunobu Nohara et al. [41]. confirmed that SHAP values offered greater interpretability than did the coefficients of generalized linear regression models, as evidenced by comparative analyses with other established interpretation methods. Using SHAP value analysis, this study identified the Rad-score and UDPS as the primary factors influencing model predictions. By quantifying the contribution of each predictor to the model’s decision-making process, SHAP analysis enhances model transparency and interpretability, ultimately facilitating a clearer understanding of the prediction mechanism.

Although this study achieved promising results, several limitations should be acknowledged. First, as a retrospective study, issues such as missing data and selection bias may have influenced the findings. Second, the sample size was relatively small, and all cases originated from a single center, which may limit the generalizability of the results. Third, the prediction model relied primarily on non-contrast CT images and lacked incorporation of multimodal data, such as laboratory tests and dual-energy CT-based urinary stone composition analysis [42]. Incorporating multimodal data may further enhance the model’s predictive accuracy. Additionally, manual segmentation of regions of interest is time-consuming and prone to inter-observer variability. The development of automated segmentation methods using deep learning could help reduce observer bias and improve reproducibility. Future studies should consider prospective designs, larger multicenter cohorts, multimodal data integration, and automated image processing techniques to enhance the robustness and clinical utility of prediction models. These improvements aim to establish more comprehensive, generalizable, and efficient tools to support medical decision-making.

## Conclusion

To increase the efficacy of ESWL for ureteral stones, minimize patient discomfort, and reduce unnecessary medical resource consumption, developing an artificial intelligence system to assist in medical decision-making is a practical and effective approach. This study integrated radiomics and conventional radiological features to develop an LR-based prediction model capable of estimating the success rate of a single session of ESWL treatment for ureteral stones. Through meticulous feature selection and multimodel comparative validation, we identified key predictive factors and provided a valuable auxiliary tool for clinical decision-making.

## Electronic supplementary material

Below is the link to the electronic supplementary material.

Supplementary Material 1

## Data Availability

The datasets used and/or analysed during the current study are available from the corresponding author on reasonable request.

## Declarations

**Human ethics and consent to participate**

In accordance with the Declaration of Helsinki, the Ethics Committee of Chongqing Western Hospital reviewed the study’s design and determined that ethical approval was not required due to its retrospective nature.

Consequently, the committee waived the need for formal ethics approval.

## Abbreviations

ESWL: Extracorporeal shock wave lithotripsy
ML: Machine learning
AI: Artificial intelligence
CT: computed tomography
UWT _max_: Maximum ureteral wall thickness
SSD: Skin-to-stone distance
UDPS: Ureteral diameter proximal to the stone
RPW: Renal pelvis width
KUB: kidneys, ureters, and bladder
ICC: Interclass and intraclass correlation coefficients
ROIs: Regions of interest
LASSO: Least absolute shrinkage and selection operator
Rad-score: Radiomics score
RF: Random forest
KNN: K-nearest neighbors
LR: Logistic regression
SVM: Support vector machine
AdaBoost: Adaptive boosting
AUC: Area under the receiver operating characteristic curve
SHAP: Shapley additive explanations
GLRLM: Gray-level run-length matrix
GLCM: Gray-level co-occurrence matrix
GLDM: Gray-level dependence matrix
PUD: Proximal ureteral diameter
