# Supplementary material for: Predicting ESWL success for ureteral stones: a radiomics-based machine learning approach
Source: BMC Med Imaging. 2025 Jul 4;25:268. doi: 10.1186/s12880-025-01817-8 (PMC12228301; doi:10.1186/s12880-025-01817-8)
Supplement: Supplementary file 1 — Supplementary Material 1 [file 12880_2025_1817_MOESM1_ESM.docx]

***Supplementary Material***

| **Features Class** | First order | GLCM | GLDM | GLRLM | GLSZM | NGTDM | Shape |
| --- | --- | --- | --- | --- | --- | --- | --- |
| Original | 18 | 24 | 14 | 16 | 16 | 5 | 14 |
| Exponential | 18 | 24 | 14 | 16 | 16 | 5 | 0 |
| Gradient | 18 | 24 | 14 | 16 | 16 | 5 | 0 |
| lbp-3D-k | 18 | 24 | 14 | 16 | 16 | 5 | 0 |
| lbp-3D-m1 | 18 | 24 | 14 | 16 | 16 | 5 | 0 |
| lbp-3D-m2 | 18 | 24 | 14 | 16 | 16 | 5 | 0 |
| logarithm | 18 | 24 | 14 | 16 | 16 | 5 | 0 |
| Square | 18 | 24 | 14 | 16 | 16 | 5 | 0 |
| Square root | 18 | 24 | 14 | 16 | 16 | 5 | 0 |
| Wavelet-HHH | 18 | 24 | 14 | 16 | 16 | 5 | 0 |
| Wavelet-HHL | 18 | 24 | 14 | 16 | 16 | 5 | 0 |
| Wavelet-HLH | 18 | 24 | 14 | 16 | 16 | 5 | 0 |
| Wavelet-HLL | 18 | 24 | 14 | 16 | 16 | 5 | 0 |
| Wavelet-LHH | 18 | 24 | 14 | 16 | 16 | 5 | 0 |
| Wavelet-LHL | 18 | 24 | 14 | 16 | 16 | 5 | 0 |
| Wavelet-LLH | 18 | 24 | 14 | 16 | 16 | 5 | 0 |
| Wavelet-LLL | 18 | 24 | 14 | 16 | 16 | 5 | 0 |

**Supplementary Table 1 | Features extracted from CT Image**

**Supplementary Table 2 |** **Prediction performance of five machine learning methods in the training and test sets**

| **Model** | **ACC（%）** | **SEN（%）** | **SPE（%）** | **PRE（%）** | **PPV（%）** | **NPV（%）** | **F1（%）** | **AUC（95%CI)** |
| --- | --- | --- | --- | --- | --- | --- | --- | --- |
| Training set (n=230) |  |  |  |  |  |  |  |  |
| RF | 0.822 | 0.869 | 0.774 | 0.794 | 0.794 | 0.856 | 0.830 | 0.893(0.853,0.929) |
| KNN | 0.791 | 0.748 | 0.835 | 0.819 | 0.819 | 0.768 | 0.782 | 0.889(0.848,0.926) |
| LR | 0.826 | 0.835 | 0.817 | 0.821 | 0.821 | 0.832 | 0.828 | 0.902(0.866,0.938) |
| SVM | 0.826 | 0.843 | 0.809 | 0.815 | 0.815 | 0.838 | 0.829 | 0.888(0.844,0.928) |
| AdaBoost | 1.000 | 1.000 | 1.000 | 1.000 | 1.000 | 1.000 | 1.000 | 1.000(1.000,1.000) |
| Test set (n=99) |  |  |  |  |  |  |  |  |
| RF | 0.798 | 0.811 | 0.782 | 0.811 | 0.811 | 0.783 | 0.811 | 0.857(0.778,0.925) |
| KNN | 0.667 | 0.604 | 0.739 | 0.727 | 0.727 | 0.618 | 0.660 | 0.767(0.675,0.854) |
| LR | 0.838 | 0.849 | 0.826 | 0.849 | 0.849 | 0.826 | 0.849 | 0.888(0.822,0.949) |
| SVM | 0.758 | 0.755 | 0.761 | 0.784 | 0.784 | 0.729 | 0.769 | 0.853(0.779,0.922) |
| AdaBoost | 0.818 | 0.849 | 0.783 | 0.818 | 0.818 | 0.818 | 0.833 | 0.881(0.802,0.943) |

***ACC* accuracy, *SEN* sensitivity, *SPE* specificity, *PRE* precision, *PPV* positive predictive value, *NPV* negative predictive value*, F1* F1 score, *AUC* area under the receiver operator characteristic curve, *CI* confidence interval**

**
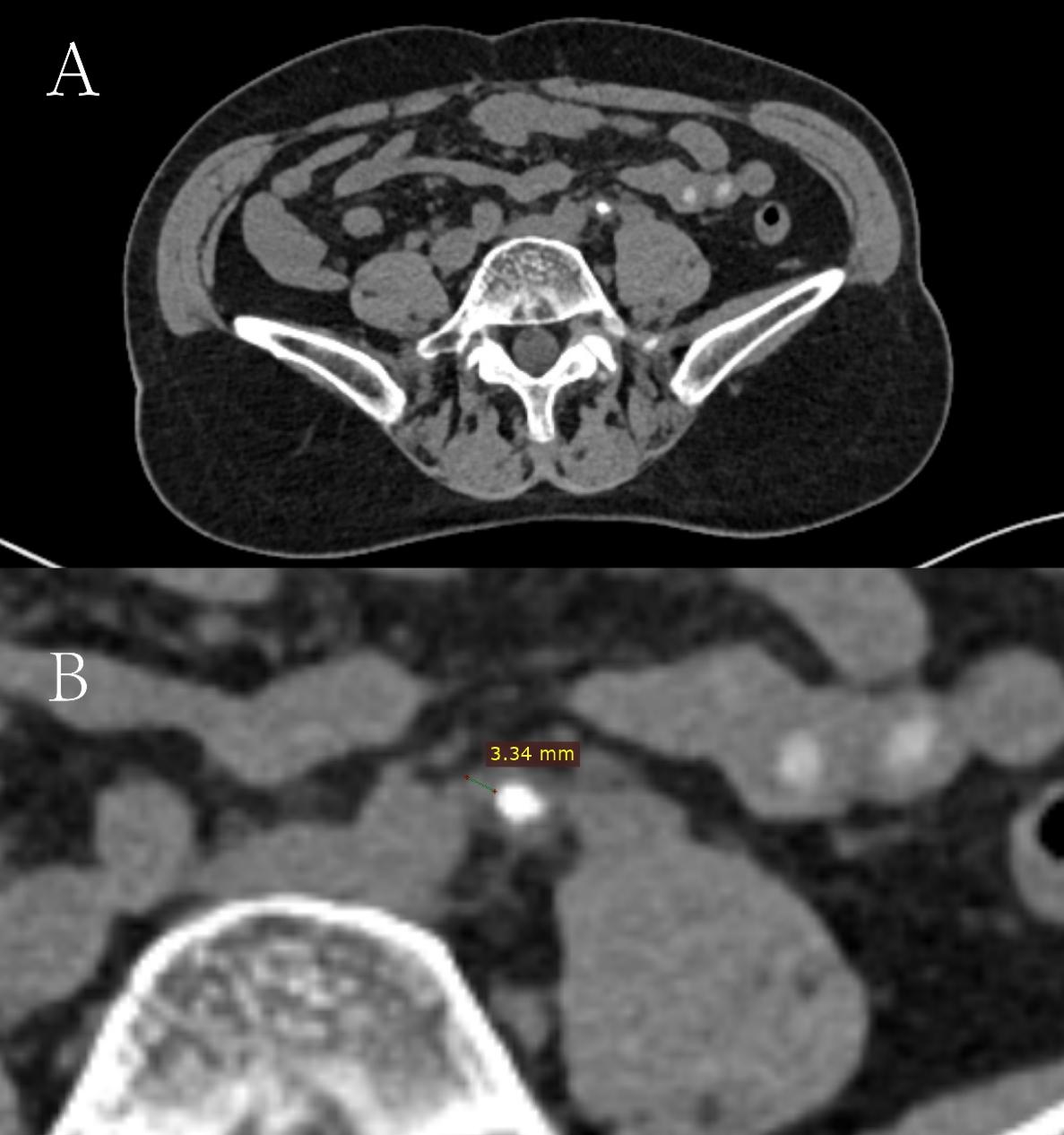
**

**Supplementary Fig. 1** UWT _max_: The maximum thickness of the ureteral wall at the stone site, measured on axial images.

**
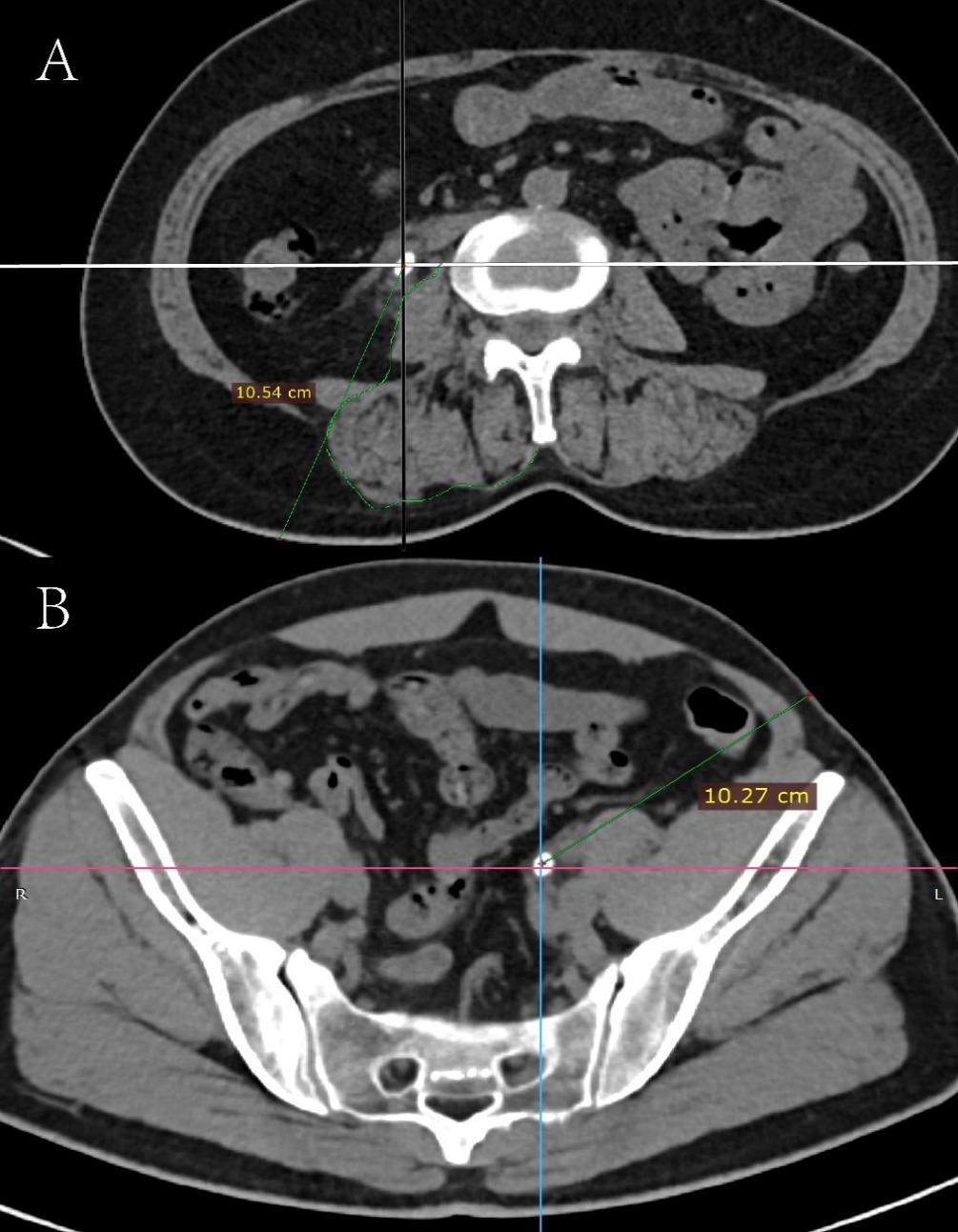
**

**Supplementary Fig. 2** SSD: The distance from the stone to the skin, measured on axial images. For the lumbar approach, SSD was measured from the stone to the skin at the outer edge of the psoas major muscle or paraspinal muscle. The abdominal approach was measured as the shortest distance from the stone to the abdominal skin.


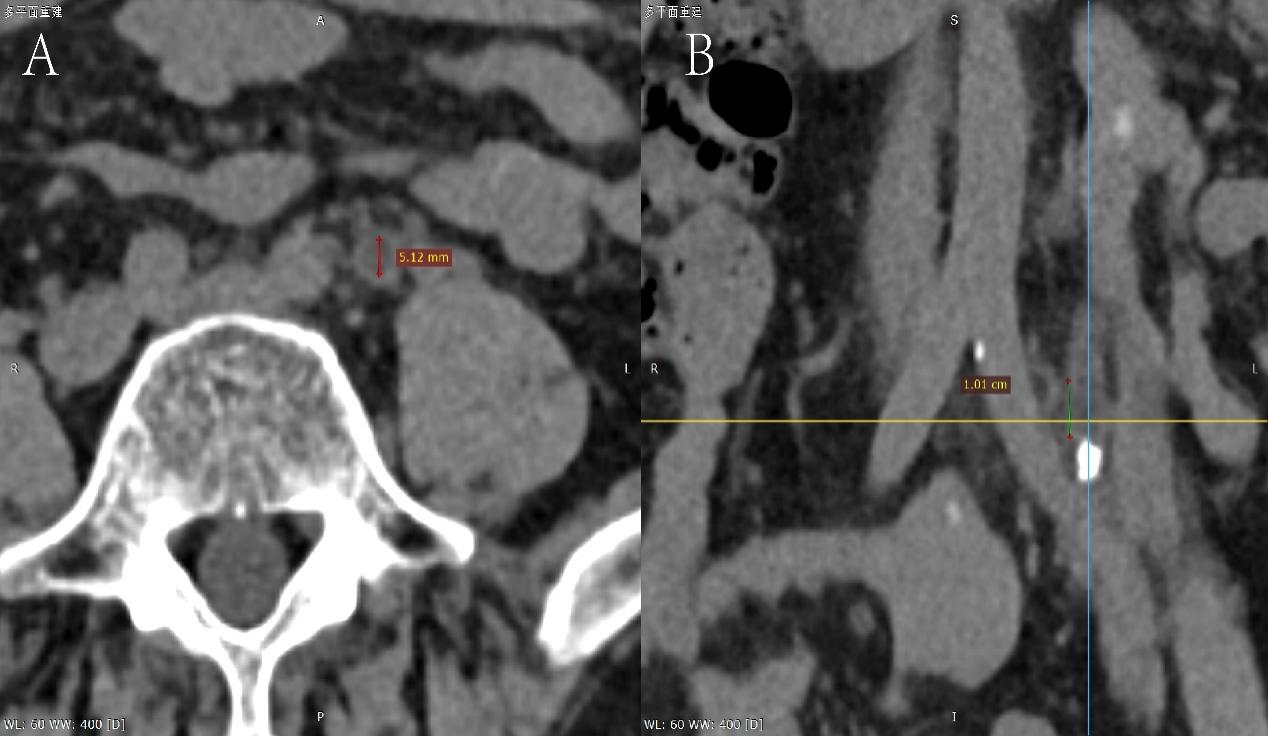


**Supplementary Fig. 3** UDPS: The widest inner diameter of the ureter within 1 cm upstream of the stone, measured on axial images in a direction perpendicular to the ureteral lumen.

**
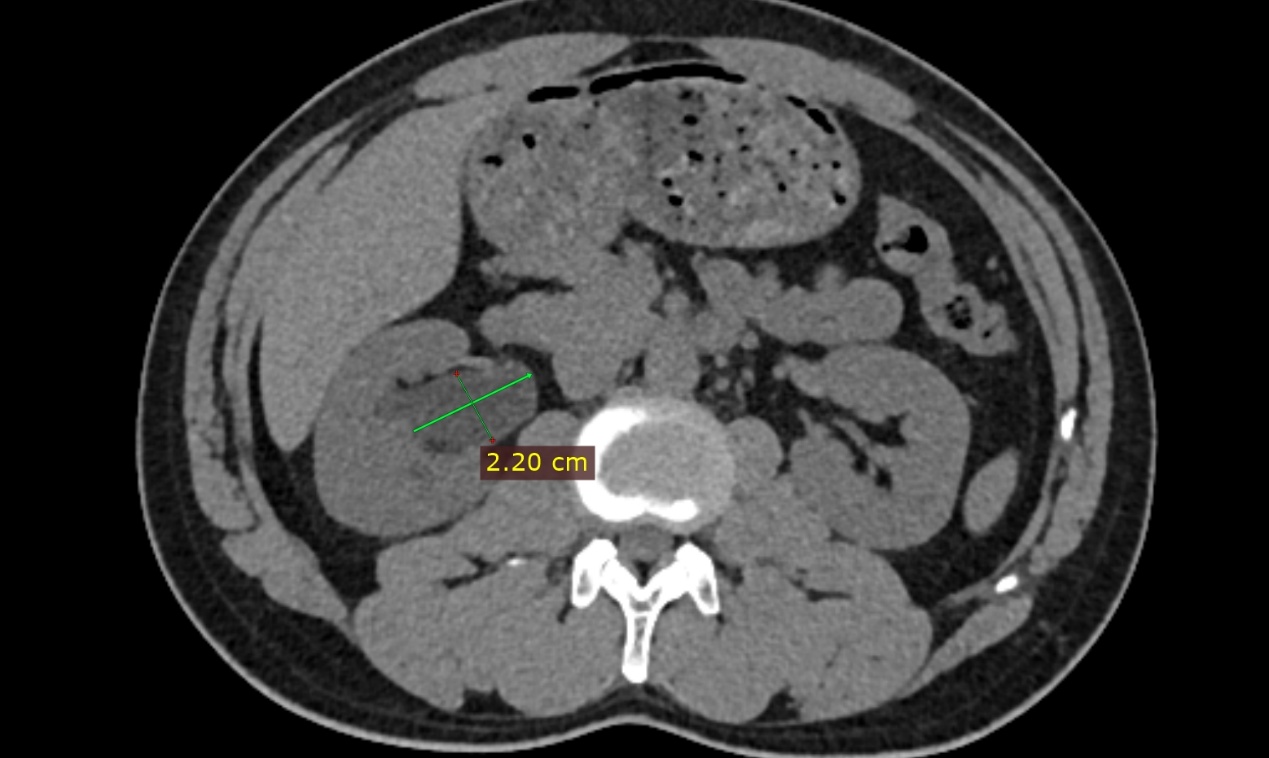
**

**Supplementary Fig. 4** RPW: The transverse diameter of the renal pelvis at its widest level, measured on axial images perpendicular to its longitudinal axis.


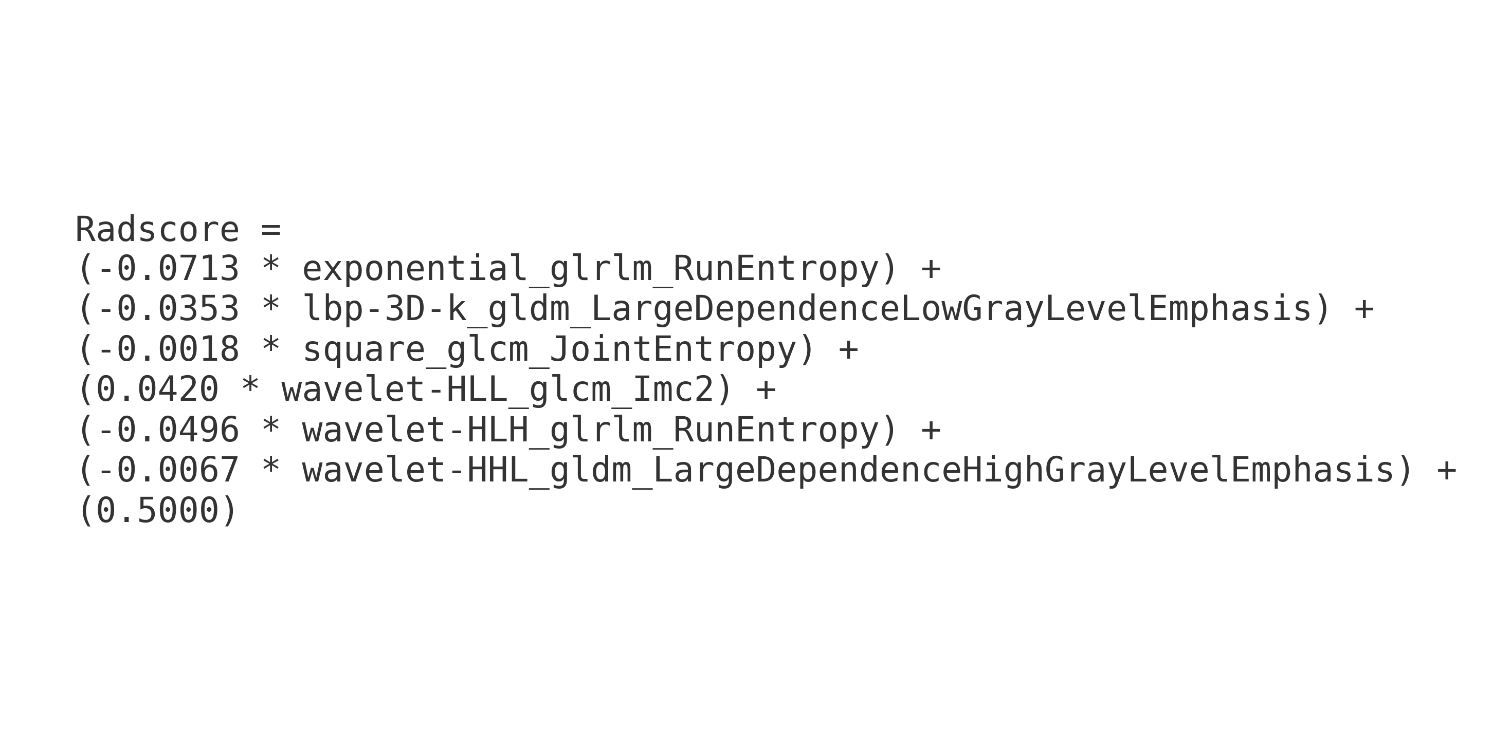


**Supplementary Fig. 5** Rad-score calculation formula.

**
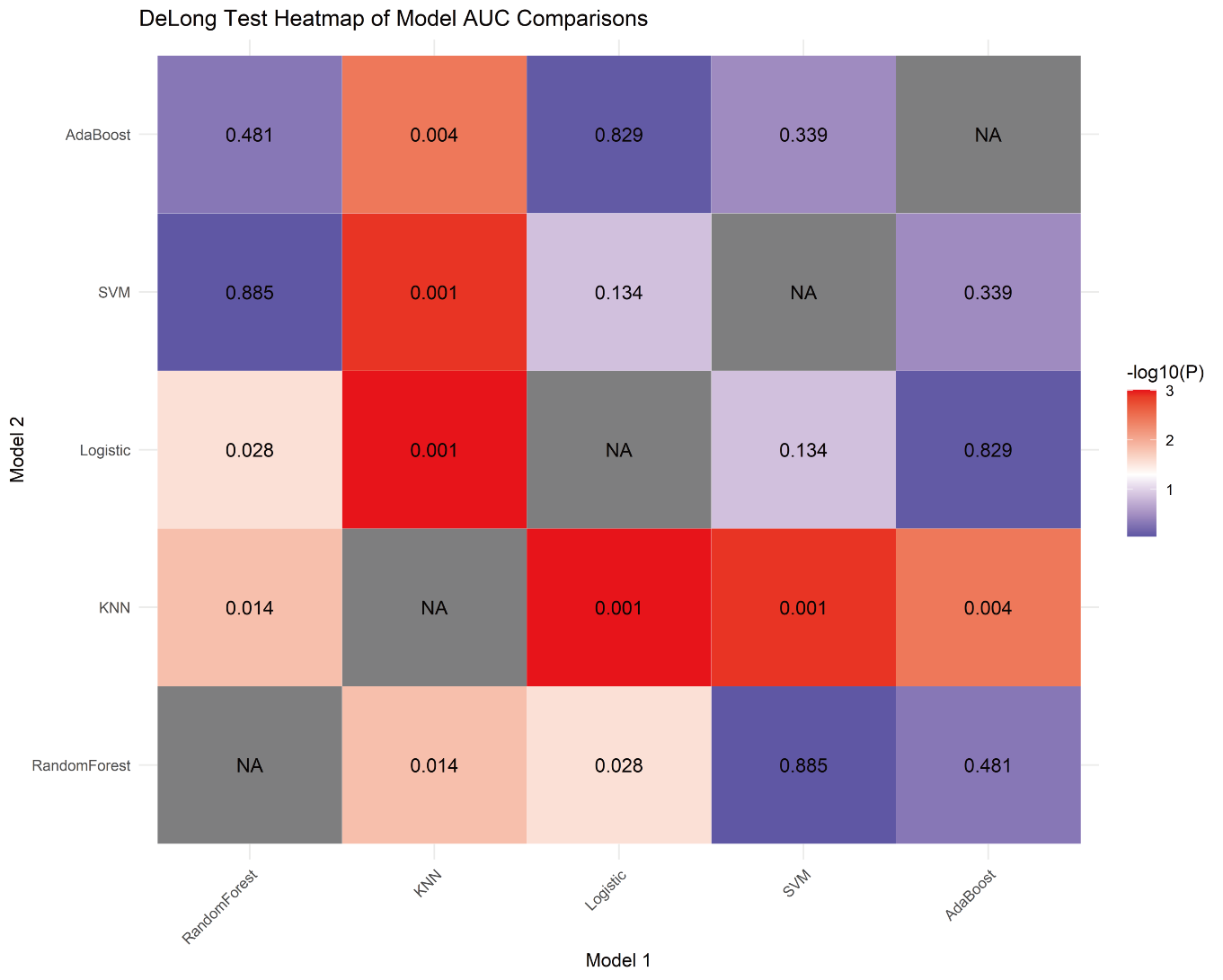
**

**Supplementary Fig. 6** Delong test heatmap of model’s AUC comparisons.

**
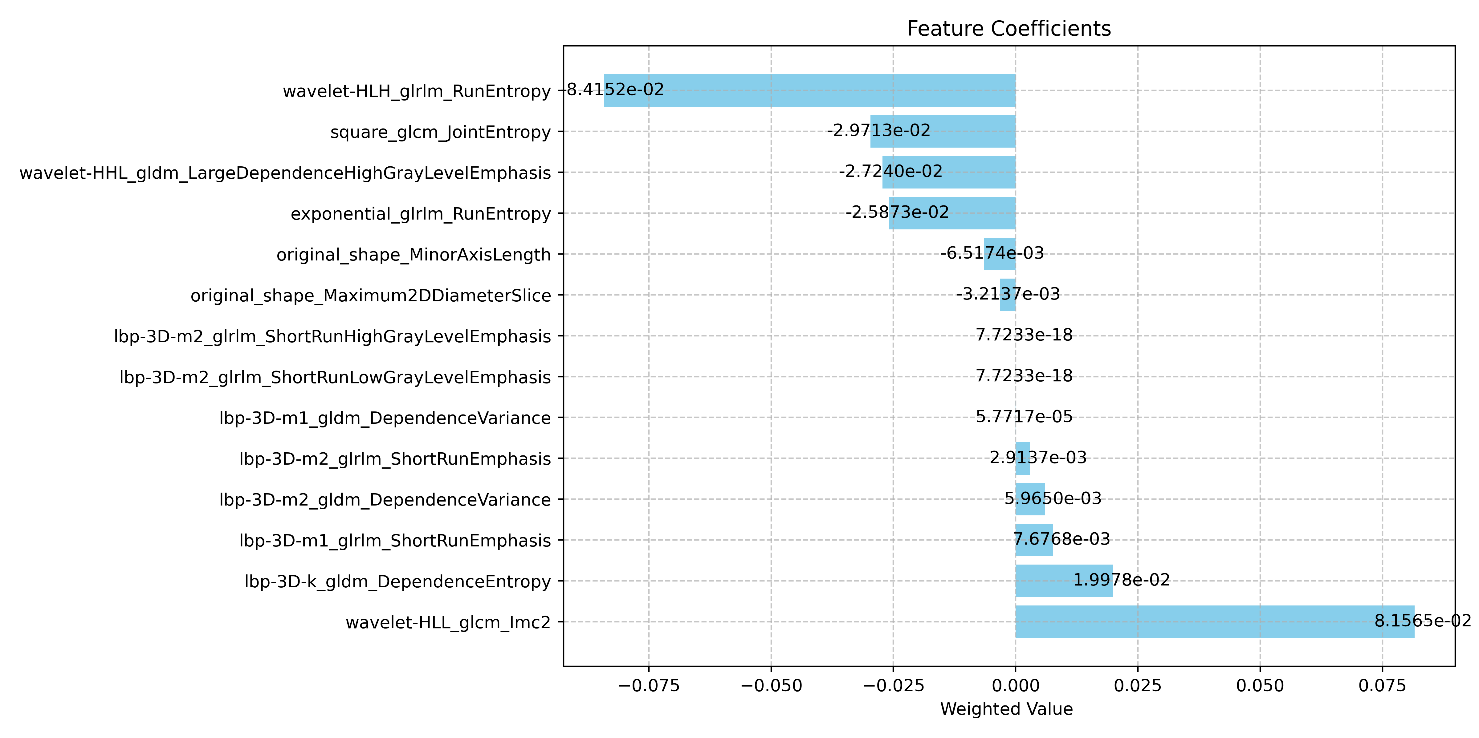
**

**Supplementary Fig. 7** 14 features selected under the λ _min_ parameter.
